# Supplementary material for: Impact of dietary patterns, individual and workplace characteristics on blood pressure status among civil servants in Bida and Wushishi communities of Niger State, Nigeria
Source: PLoS One. 2019 Dec 13;14(12):e0226231. doi: 10.1371/journal.pone.0226231 (PMC6910664; doi:10.1371/journal.pone.0226231)
Supplement: Attachment — Submitted filename: Response to Reviewer comments.docx [file pone.0226231.s002.docx]

|  | Reviewer’s Comments | Response to Reviewer’s Comments |
| --- | --- | --- |
|  | Title section: probably would need to exclude "Factor and logistic regression analyses" | "Factor and logistic regression analyses" has been excluded |
|  | Abstract section:  It is recommended not to use abbreviations in this section.  In addition, you said that this problem has not been explored in Nigeria, how can you be sure that?  Take home message should be added in conclusion | Abbreviation used had at first appearance been defined in full.  The word “fully” been added  These words were inserted:  “The prevalence of hypertension among the participants in this study is relatively very high. Increase in age and working in educational sector are risk factors associated with hypertension. Therefore, it is recommended that civil servants engage in frequent exercises and have medical checkup more regularly especially as one gets older” |
|  | Introduction section: 1. Page 3 (Line 65, 72), please correct as SSA, provide their full text first for SBP and DBP | SSA was corrected  Full text for Systolic Blood Pressure (SBP) and Diastolic Blood Pressure (DBP) were inserted |
|  | Page 3, 4. Please rewrite the sentence beginning with between blood pressure and end with BMI (Line, 82, 83) | Sentence was corrected as:  Significant associations between blood pressure variables, weight and Body Mass Index (BMI) have also been reported in several studies (16,17) |
|  | Page 4, Line 91. Where is elsewhere? | “and elsewhere” remove  Corrected as “American and China” |
|  | Page 5, Line 102-107. Taking about factor analyses, I think this paragraph should be stated in methodology section | The paragraph was moved to methodology section |
|  | . In general, when we state the introduction section, we have to state in the following way;  General description of the problem under study including its meaning, burden/magnitude of the problem from global to local and from past to current, whose is affected, where, when, the consequences of the problem, causes, what has been done before by the Government of Nigeria to reduce this problem, factors attributable of the problem identified by previous research works | Most of what are required from here for introduction are already contained in it. For those we could not provide are because we could not lay hands on right information |
|  | Methods section: 1. Page 5, Line 115. Description of the study setting and design were not included | These we have added and correctly given heading where it was not given before |
|  | . Page 6, Line 122. Describing about DDF is better if you move it in to variables measurement and operational definition section | This has been moved to the appropriate section as recommended |
|  | . Page 7. You estimated a final sample size of 440, then, why you included 530 participants. Is your sampling technique being cluster sampling, if so, you need to account the design effect while calculating your sample size? | ‘design effect 2’ was added |
|  | Many sections of the methodology were not appeared. Sampling procedure, study population, exclusion and inclusion criteria, variable measurements, operational definition, and others. Please follow the scientific paper writing format as others did | Though many of what is requested are already in the paper, but looped up into one heading. Based on this observation the have been extracted and placed under appropriate heading. Those missing have also been added |
|  | Where were your data collectors, their profession and educational background, the language used to prepare the data collection tool | This statement was added: “The survey was conducted over a period of 6 weeks (from 3rd July to 14th August, 2018) by the 4 researchers and assisted by 3 undergraduate students. English language was the medium of communication during the survey. However, where it becomes necessary to explain to the participant in local dialects, this was also done |
|  | Define others in respective tables (Table, 1, 2). | Definition of ‘others’ in table 1 is defined under variable measures and operational definition section as:  (these include any MDAs that could not be classified among the ones identified), while for other places in table 2, it has been replaced with appropriate variable names |
| **In addition: Response to Editor Comments** | | |
| 1 | To provide a short title | Short title has been added |
| 2 | The in-text reference bracket be changed to square bracket | This has been done using the referencing manager |
| 3 | It is not quite clear where one variable ends and the other starts. Please, kindly add a semi-colon between them. | Semi colon has been used to separate them |
| 4 | Please, add a reference to what this symbol means | The meaning of the symbol has been added |
| 5 | Please, add reference to what this ref means | The meaning of ref has been added |
| 6 | Please note that if you wish to use the structure: *… the findings reported in (XX)…*, you need to cite the name of the author of the report | The names of the authors have been added |
| 7 | Please, consider mentioning this under the Methods section: “ no multiple measures of BP taken except when SBP was “if the readings of SBP were outside the range of 80-mm Hg and 240-mm Hg and DSP were less than 30-mm Hg, in which case the measure was repeated after a 5 minutes interval to validate the reading” | I think it is okay were it is stated as limitation of study. It better to keep the reader in suspense till that later part of the work. |
|  | This sentence is not quite clear. Do you mean that no participant was excluded, even if they did not complete the health status section? | I think the sentence is very clear: “no participant was excluded on the basis of having health status that could modify…” |
|  | This sentence is not entirely clear. Do you mean the authors state that the results should not be generalized to other areas of the Country? | Yes, because of the limitations established, restrain could be taking by the readers from generalizing the results |
|  | Please, check whether you need to acknowledge people or support | This has already been submitted to the journal through a separate file |
|  | You will find two reference lists below. The first is an unlinked list that I have manually formatted to include all the required changes, since we do not use reference management software. Please use my list as reference and remember to make the suggested changes in the settings of the software program to incorporate them into your software-generated reference list (which is the second list). Once this is done, please delete the list that I prepared | Action taken appropriately. However, some papers do not have pagination on it (1 or 2 of them) |
